# Transcriptome analysis of the hormone-sensing cells in mammary epithelial reveals dynamic changes in early pregnancy

**DOI:** 10.1186/s12861-015-0058-9

**Published:** 2015-01-27

**Authors:** Duvini De Silva, Kamini Kunasegaran, Sujoy Ghosh, Alexandra M Pietersen

**Affiliations:** Laboratory of Mammary Gland Biology, National Cancer Centre Singapore, 11 Hospital Dr, Singapore, 169610 Singapore; Program in Cancer & Stem Cell Biology, Duke-NUS Graduate Medical School, 8 College, Rd, 169857 Singapore, Singapore; Program in Cardiovascular & Metabolic Disorders, Duke-NUS Graduate Medical School, 8 College Rd, Singapore, 169857 Singapore; Department of Physiology, National University of Singapore, 21 Lower Kent Ridge Rd, Singapore, 119077 Singapore

**Keywords:** Mammary gland, Morphogenesis, Single cell analysis, Proliferation, Estrogen receptor, Microarray

## Abstract

**Background:**

Alveoli, the milk-producing units of the mammary gland, are generated during pregnancy by collaboration of different epithelial cell types. We present the first analysis of transcriptional changes within the hormone sensing population during pregnancy. Hormone-receptor positive (HR+) cells play a key role in the initiation of alveologenesis as they sense systemic hormonal changes and translate these into local instructions for neighboring HR- cells. We recently showed that IGF2 is produced specifically by HR+ cells in early pregnancy, but is undetectable in the virgin state. Here, we define the transcriptome of HR+ cells in early pregnancy with the aim to elucidate additional changes that are unique for this dynamic developmental time window.

**Results:**

We harvested mammary glands from virgin, 3-day and 7-day pregnant mice and isolated a few hundred hormone-sensing cells per animal by FACS for microarray analysis. There was a high concordance between animals with a clear induction of cell cycle progression genes at day 3 of pregnancy and molecules involved in paracrine signalling at day 7.

**Conclusions:**

These findings underscore the proliferative capacity of HR+ cells upon specific stimuli and elucidate developmentally-restricted changes in cellular communication. Since the majority of breast cancers are HR+, with a variable proportion of HR+ cells per tumor, we anticipate that this data set will aid further studies into the regulation of HR+ cell proliferation and the role of heterotypic signalling within tumors.

**Electronic supplementary material:**

The online version of this article (doi:10.1186/s12861-015-0058-9) contains supplementary material, which is available to authorized users.

## Background

The adult mammary gland of the mouse contains a branching structure of epithelial milk ducts embedded in the mammary fat pad. The epithelial ducts are bi-layered; the outer basal layer consists mainly of contractile myoepithelial cells and the luminal layer contains both hormone receptor positive (HR+) and negative (HR-) cells. HR+ cells are identified by their expression of the steroid hormone receptors for estrogen and progesterone (ER and PR) [1], and they also have a high expression of the prolactin receptor [2]. Luminal HR- cells are characterised by expression of the transcription factor Elf5 and already express low levels of milk genes even in the virgin state [2,3]. In the adult virgin epithelial cells rarely proliferate, but the ones that do are usually luminal HR- cells [4,5]. In *in vitro* assays, HR- cells form colonies whereas the majority of HR+ cells are non-clonogenic [6]. Together, this has led to the concept that HR+ cells are relatively mature, or terminally differentiated, cells [7,8]. However, Ewan and colleagues showed that TGFbeta signaling is actively required to prevent proliferation by HR+ cells [9] and another report documented a 10-fold increase in proliferating HR+ cells in early pregnancy [10]. Interestingly, a study that used ovarectomized mice treated with hormone injections to mimic early pregnancy in a time-controlled manner showed that there is a short first wave of proliferation of HR+ cells, followed by a larger wave of proliferation of HR- cells [11].

Upon pregnancy, there is increased branching of the milk ducts on which lobular structures of alveoli (future sites of milk production) are formed [1]. HR- luminal cells are molecularly primed for milk production and as such are referred to as alveolar progenitor cells. However, these progenitor cells do not provide all the progeny that generate the alveoli. Recent data by others and us showed that alveologenesis occurs to a large extent by collaborative outgrowth of the three main epithelial cell lineages; basal cells and luminal HR+ and HR- cells [12-14]. This is consistent with an important role for cellular communication in alveolar development [15].

Pregnancy causes an increase in progesterone and prolactin levels and both these hormones are required for the initiation of alveologenesis [1]. HR+ cells translate these systemic hormonal signals into local instructions for neighboring cells by paracrine signaling. For instance, progesterone and prolactin induce expression of RANKL [2,16], a growth factor that is essential to induce proliferation of neighboring HR- cells [11]. In addition, we found that another growth factor that is essential for alveologenesis, IGF2 [17], was produced specifically by HR+ in early pregnancy [2]. Notably, IGF2 is undetectable in virgin state [2] and therefore we wondered what other factors these cells produce specifically during active morphogenesis in early pregnancy.

Here, we analyzed the transcriptome of HR+ cells at two early time points in naturally-induced pregnancy to characterize these cells in a state of active proliferation and cellular communication.

## Results and discussion

### Pregnancy induces proliferation in both HR+ and HR- cells

**Figure 1:**
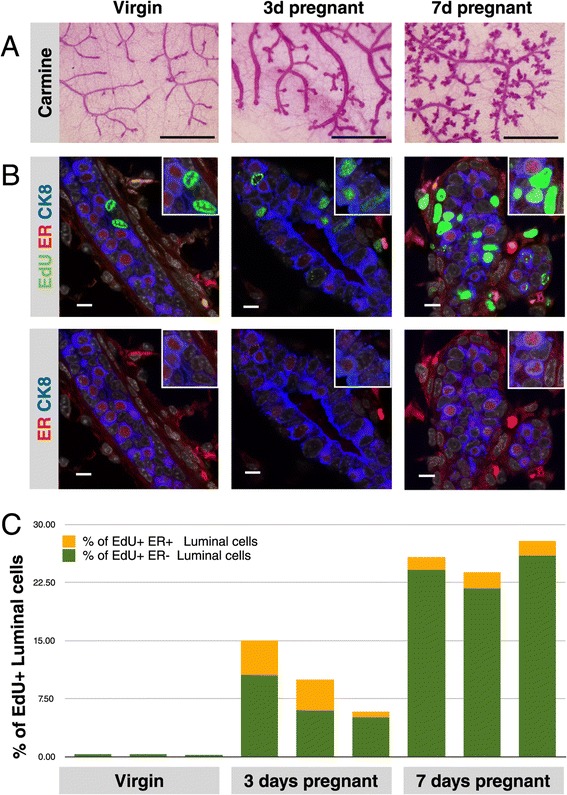
**Pregnancy induces proliferation in both the HR+ and HR- cells. (A)** Representative images of carmine-alum-stained whole mounts of mammary glands from virgin, 3-day and 7-day pregnant FVB/N mice. Scale bar, 1 mm. **(B)** Confocal immunofluorescence of mammary gland sections from virgin, 3-day and 7-day pregnant mice stained for the proliferation marker 5-ethynyl-2’-deoxyuridine (EdU, green), Estrogen Receptor (ER, red) and the luminal cell marker cytokeratin-8 (CK8, blue). Scale bar, 10 μm. The exposure time for the ER signal was increased in pregnant samples to allow robust identification of ER+ cells (see Additional file 3 for images with a fixed exposure time). **(C)** Quantification of the proportion of luminal cells that is proliferating, separated by cell type based on ER expression. ER+ and ER- luminal cells start proliferating at day 3 of pregnancy, by day 7 the majority of proliferating luminal cells are ER-. Bars represent individual animals.

To characterize the changes that occur in HR+ cells in early pregnancy, we obtained mammary glands from FVB/N mice that were adult virgins (nulliparous), and from timed-mated mice at day 3 and day 7 of pregnancy. Carmine staining of the thoracic mammary glands confirmed the presence of relatively bare milk ducts at the virgin state (metestrus), increased branching and thickening of the ducts at day 3 of pregnancy and the appearance of alveolar structures by day 7 of pregnancy (Figure 1A). We evaluated the proliferative status of the HR+ cells by EdU injection 24 hours before harvest. Paraffin sections were stained with antibodies against cytokeratin 8 (CK8, blue) to identify luminal epithelial cells and the estrogen receptor (ER, red) as a marker for HR+ cells. In this case, we chose ER to identify HR+ cells but it is important to note that not all ER+ cells co-express the progesterone receptor (PR) and *vice versa* [5]. This can be due to receptor downregulation upon active signaling [18] but potentially could also indicate a further heterogeneity within the HR+ cell population [19]. Similar to previous literature [4,10], we found that in mammary epithelium not many epithelial cells are proliferating in the virgin state, and the rare cells that do are all ER- (Figure 1B). Pregnancy induced considerable proliferation of luminal epithelial cells, including the ER+ cells (Figure 1B and C). By day 7 of pregnancy, the proportion of proliferating luminal cells that are ER+ diminishes, whereas the proportion of proliferating ER- luminal cells (that are primed for milk production) continues to increase (Figure 1C). Beleut and colleagues demonstrated that steroid hormone injections first induced proliferation in a portion of progesterone receptor positive (PR+) cells, followed by proliferation in a much larger proportion of PR- cells [11]. Our data show that this response also occurs during a natural pregnancy, in which ER + HR+ cells proliferate for a brief initial period, whereas the luminal HR- cells also start proliferating early on, but continue to expand considerably during the course of pregnancy.

### Identification of HR+ cells by cell surface markers in early pregnancy

**Figure 2:**
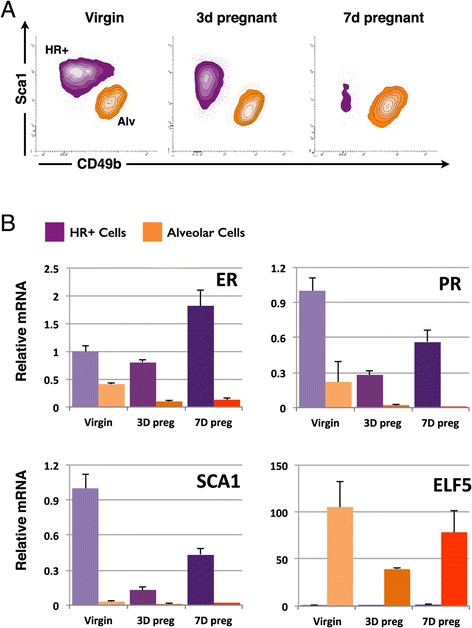
**Purification of HR+ cells by FACS in early pregnancy. (A)** The luminal population of mammary epithelial cells (CD24^hi^CD49f^lo^) was separated into hormone-sensing cells (Sca1^hi^ CD49b^lo^; purple gate) and alveolar progenitor cells (Sca1^lo^ CD49b^hi^; orange gate). **(B)** Quantitative RT-PCR on 500 directly-lysed cells per sample for markers that distinguish the two luminal cell types. Values are relative to mRNA levels in HR+ cells from virgin animals and normalised to HPRT expression. Error bars indicate standard deviation for 3 individual mice.

To identify molecular changes during the burst of proliferation of HR+ cells, we isolated HR+ epithelial cells from the abdominal mammary glands of virgin mice at metestrus and 3-day and 7-day pregnant mice. Single cells were stained with cell surface markers and isolated by FACS. After exclusion of doublets, dead cells and lymphocytes, luminal epithelial cells were identified by their high expression of CD24 and relatively low expression of alpha6-integrin (CD49f, see gating strategy in Additional file 1 and 2). The luminal cell population was separated into HR+ and HR- cells based on Sca1 (Ly6A) and alpha2-integrin (CD49b) expression (Figure 2A). The proportions of the various FACS populations for individual animals can be found in Additional file 2. Notably, Sca1 expression went gradually down in HR+ cells upon pregnancy. Due to the continued proliferation of the luminal HR- cells, the relative proportion of HR+ cells at day 7 of pregnancy is considerably smaller compared to the virgin state (Figure 2A). To ensure that the cell surface profiles in pregnancy still identified the HR+ population despite the changes in Sca1 expression, the two distinct populations were sorted for qPCR analysis. We made use of a direct lysis method we recently developed for mRNA analysis of small numbers of cells [20] and 500 cells per population were analyzed from three individual mice. There is some fluctuation in hormone receptor transcription during pregnancy (Figure 2B), which could be due to changes in transcriptional activity but also potentially to alterations in cellular subsets within the HR+ population [19]. Note that ER protein levels go down in early pregnancy (Additional file 3) but ER transcript levels do not. Because ligand binding induces hormone receptor degradation [18] the relation between transcript and protein levels is not straight forward. Nevertheless, this qPCR validation shows that the CD49f^lo^ population clearly contains the HR+ cell population despite the reduction in Sca1 expression. In addition, the HR- population is identified by the expression of Elf5 and this population does not contaminate the HR+ population sorted based on Sca1 and CD49b (Figure 2B).

### Transcriptome analysis of HR+ cells obtained in early pregnancy

**Figure 3:**
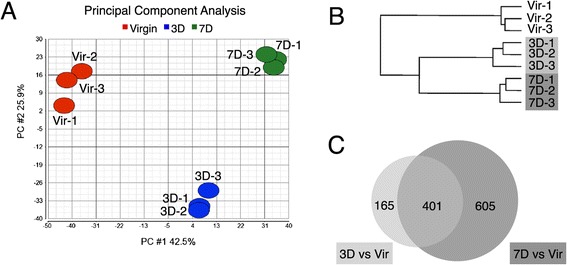
**Transcriptome analysis clusters HR+ cells by developmental state. (A)** Principal Component Analysis (PCA) was performed on three biological replicates of HR+ cells derived from virgin (Vir), 3-day (3D) and 7-day (7D) pregnant mice based on 19,821 probes (see Methods for details). A scatterplot of the first two principal components demonstrates a separation by pregnancy along the first principal component (PC1) and by duration of pregnancy along the second principal component (PC2). **(B)** Unsupervised hierarchical clustering based on 1328 probes (probes with an absolute fold-change > =2 and false discovery rate (FDR) of 10% or less) shows that the biological replicates cluster according to developmental state. **(C)** Venn diagram illustrating the number of genes that were changed more than 2-fold with a FDR of 10 in the 3 replicates of HR+ cells isolated at day 3 of pregnancy (3D, light grey) compared to virgin samples and the overlap of this gene set compared to differentially expressed genes at day 7 of pregnancy (7D, dark grey, more than 2 fold change compared to virgin).

To carry out a comprehensive gene expression analysis of the changes that occur in HR+ cells in early pregnancy, we adapted our direct lysis protocol for microarray analysis. In this case, we sorted a thousand HR+ cells per animal and used an aliquot equivalent to 167 cells for RNA amplification and subsequent hybridization to Illumina arrays. Note that this cell number is low enough to allow sampling from individual mice even when the cellular subset is small, while the cell number is large enough to provide a robust average of the population without the need for many repeats, as would for instance be the case with single cell analysis. The assumption that a few hundred cells per population provides a consistent representation of the changes that occur within the HR+ cell population is supported by the high reproducibility of the transcriptional profiles of samples taken from individual animals (n = 3 animals per time point), as indicated by a principal component analysis (Figure 3A). Unsupervised hierarchical clustering also confirms that the HR+ populations of the different time points are most similar to samples of the same developmental stage (Figure 3B).

At day 3 of pregnancy, there is a set of 165 genes that have changed more than 2 fold compared to the virgin samples and that are distinct from changes that occur at day 7 of pregnancy. There is a larger group of 401 genes that is changed at both the 3-day and 7-day time point, in addition to 605 genes whose expression only starts changing at day 7 of pregnancy (Figure 3C, genes listed in Additional file 4).

**Figure 4:**
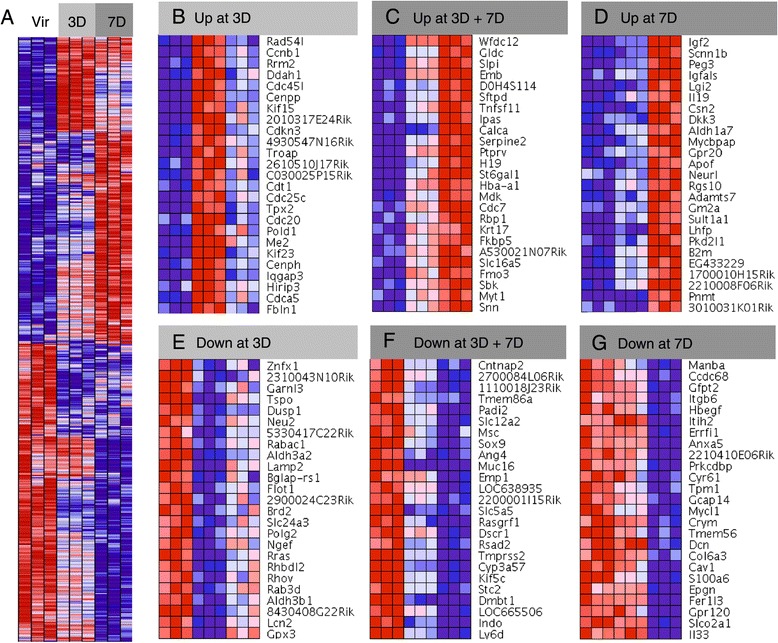
**Heat maps of transcriptional changes in HR+ during early pregnancy. (A)** Heat map of Illumina gene probes generated by GenePattern software showing transcriptional changes during early pregnancy. **(B-C)** Heat maps generated with an input of the 25 most up-regulated and 25 most down-regulated genes in each category based on the values of their Pi scores (see Methods). Twenty-five gene probe sets that are induced **(B)** and reduced **(C)** most strongly in HR+ cells specifically at day 3 of pregnancy. **(D-E)** Gene probe sets that are most strongly induced **(D)** and reduced **(E)** in early pregnancy. **(F-G)** Gene probe sets that are induced **(F)** and reduced **(G)** most strongly in HR+ cells specifically at day 7 of pregnancy.

Figure 4A illustrates that distinct gene clusters can be recognized based on the direction of the transcriptional changes. For instance, there are genes that are strongly upregulated at day 3 but are reduced again at day 7 (Figure 3B), genes that are induced gradually from day 3 to day 7 (Figure 3C), or become induced only by day 7 (Figure 3D). Similar trends can be observed for downregulation of genes, in which a cluster of genes is abruptly downregulated at day 3 (Figure 3E), but there are also genes whose expression gradually decreases with the progression of pregnancy (Figure 3F) and some genes are only downregulated by day 7 (Figure 3G).

### Response of HR+ cells at day 3 of pregnancy is characterized by proliferation and changes in hereditary breast cancer pathways

**Figure 5:**
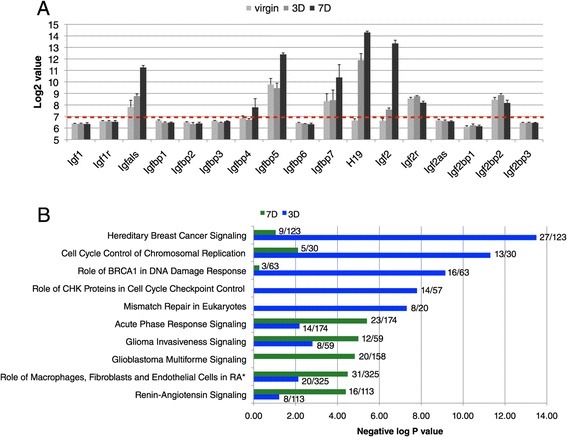
**Pathway analysis of changes in HR+ cells during early pregnancy. (A)** Bar chart of the transcriptional changes of genes involved in IGF signaling. Values are normalized Log2 values of Illumina probe sets, error bars denote standard deviation of three biological replicates. The dotted red line indicates background noise. **(B)** Ingenuity Pathway Analysis showing canonical pathways that are most significantly changed at day 3 of pregnancy (blue) and at day 7 of pregnancy (green) compared to virgin samples. The number of genes in each pathway that was significantly changed in the HR+ samples is indicated at the top of each bar.

In our previous work, we noticed that IGF2 transcription is strongly induced in HR+ cells in early pregnancy [2]. Analysis of the microarray data shows that additional players in the IGF signalling network are also induced (Figure 5A), such as Insulin-like growth factor acid labile subunit (IGFALS) which increases the half-life of IGFs [21] and also two Insulin-like growth factor binding proteins (IGFBP5 and IGFBP7) that have opposing roles in mammary gland involution [22,23]. The long non-coding RNA H19, whose transcription is tightly linked to that of IGF2 [24], is strongly induced already at day 3 of pregnancy (Figure 5A). This seems earlier than the induction of IGF2, but that could also be due to differences in detection sensitivity of the array. Both IGFBP5 and H19 have been suggested to antagonise IGF signalling and therefore these data suggest an intricate regulation of IGF signalling in mammary epithelium in early pregnancy.

An unbiased examination of changes in signalling pathways was performed by Ingenuity Pathway analysis. The pathways that were most significantly changed in HR+ cells at day 3 of pregnancy compared to the virgin samples comprised pathways related to the cell cycle and DNA repair check points (Figure 5B). Interestingly, several of the transcriptional changes at day 3 occur in genes that have been implicated in hereditary breast cancer, such as p53, Chek2 and FANCD2. A complete list of the 27 genes that are responsible for the significant change in the Hereditary Breast Cancer Ingenuity Pathway is included in Additional file 5. At day 7 of pregnancy, changes grouped according to Ingenuity signalling pathways were less significant and occurred to some extent already at day 3 (Figure 5B). These pathways were mostly related to immune function and cell migration and included genes such as integrins and PI3KR3, that were for instance assigned to the Ingenuity pathway ‘invasive glioma signaling’.

**Figure 6:**
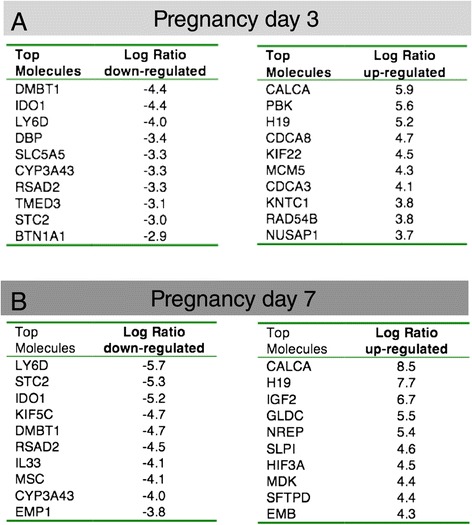
**Top molecules that are differentially expressed in HR+ cells in early pregnancy. (A)** Molecules identified by Ingenuity Pathway Analysis that are most strongly down- and upregulated in HR+ cells at day 3 of pregnancy compared to virgin samples. **(B)** Molecules identified by Ingenuity Pathway Analysis that are most strongly down- and upregulated in HR+ cells at day 7 of pregnancy compared to virgin samples.

The individual genes whose transcription is changed most dramatically in HR+ cells in early pregnancy are listed in Figure 6. For instance, at day 3 of pregnancy several of the most highly induced genes are involved in proliferation (Figure 6A). This includes cell cycle genes (CDCa3 and CDCa8) and mitotic genes such as PDZ-binding kinase (PBK) [25], Kinesin-like protein 22 (Kif22), Kinetochore associated 1 (KNTC1), Nucleolar and spindle-associated protein 1 (Nusap1). Minichromosome maintenance complex component 5 (MCM5) is a DNA replication licensing factor [26] and RAD54B plays a role in homologous recombination and repair of DNA [27]. Additional file 6 contains a summary of genes involved in the cell cycle that are transcriptionally changed in early pregnancy.

Other top upregulated genes at day 3 of pregnancy are known targets of steroid hormones, such as calcitonin (CALCA) and IGFALS [28,29]. The genes that are strongly down regulated at pregnancy day 3 are mostly similar to the genes that are down regulated at day 7 (Figure 6B) and they do not cluster into obvious functional groups. Indoleamine 2,3 dioxygenase (IDO1), an enzyme that catabolizes trypthophan, has a potentially immunosuppressive role and high IDO expression in ER+ breast cancer is associated with a better overall survival [30,31]. D site of albumin promoter (albumin D-box) binding protein (DBP) is a transcription factor that for instance binds to the insulin promoter. The sodium/iodide transporter (SLC5A5) is negatively regulated by IGF-1 and TGF-beta signalling in the mammary gland [32] and therefore its down regulation may be a reflection of increased IGF signalling.

**Figure 7:**
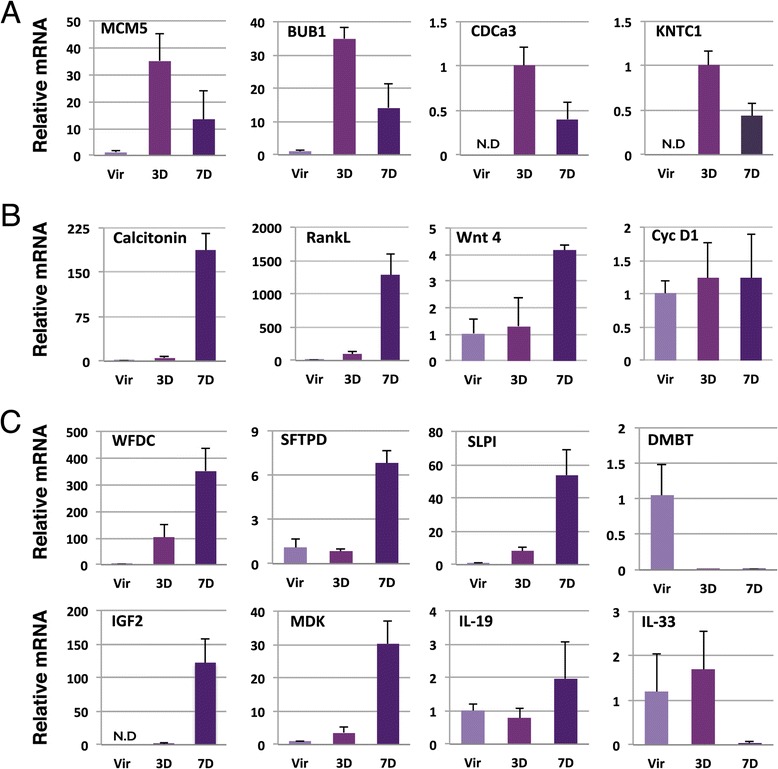
**Quantitative polymerase chain reaction (qPCR) validation of the transcriptional changes in HR+ in early pregnancy. (A)** Relative mRNA expression of cell cycle genes during pregnancy compared to virgin samples, normalised to HPRT expression. **(B)** Relative mRNA expression of progesterone target genes during pregnancy compared to virgin samples, normalised to HPRT expression. **(C)** Relative mRNA expression of secreted factors during pregnancy compared to virgin samples, normalised to HPRT expression. N.D.: Not Detected. Error bars indicate standard deviation for 3 individual mice.

Taken together, at day 3 of pregnancy the most striking change in the transcriptome of HR+ cells compared to HR+ in the non-pregnant mammary gland is the induction of proliferation. This is indicated by the Ingenuity Pathway Analysis, the individual genes that change most significantly based on the microarray analysis, and this was further validated by qPCR on some of the cell cycle genes on independent cDNA samples (Figure 7A).

### Transcriptional changes in HR+ cells at day 7 of pregnancy involve immune regulation and cellular communication

The expression of cell cycle progression genes is still apparent at day 7 of pregnancy, but to a lesser extent. This is consistent with the initial wave of proliferation in HR+ cells that is superseded by proliferation in the luminal HR- cells (Figure 1). Part of the overlap in gene changes at pregnancy day 3 and day 7 (Figure 3C) is due to the induction of cell cycle genes, but there are other processes that partially overlap that are only just detectable at day 3 and become much more robust at day 7. For instance, the induction of progesterone targets calcitonin and RANKL is detectable at day 3 of pregnancy, but the fold induction is much higher at day 7 (Figure 7B). Another target of the progesterone receptor (PR), Wnt4, is not detectable with the relatively low sensitivity of the microarray but an increase in Wnt4 expression at day 7 of pregnancy can be detected by qPCR (Figure 7B). Cyclin D1 has also been found to be partially regulated by PR [33] but we do not find evidence for Cyclin D1 induction either by microarray or qPCR (Figure 7B). Induction of cyclin D1 transcription was described 24 hours after progesterone injection, but in that case RNA from the entire mammary gland was used [34], implying that the cyclin D1 induction could have occurred in neighboring HR- cells.

In addition to elevated cell cycle genes and known hormone-induced target genes, this data set provides insight to other processes that are initiated in HR+ cells at day 7 of a murine pregnancy, the time when alveoli become clearly visible (Figure 1A). In Figure 7, we highlight a few of the top molecules that are all secreted factors (with the exception of Cyclin D1). A complete list of transcriptional changes in secreted factors (annotation by Ingenuity) is included in Additional file 7.

WFDC (WAP four-disulfide core domain) is a protease inhibitor with a potential role in immune regulation. Interestingly, WFDC is part of the same gene cluster as SLPI (Secretory Leukocyte Protease Inhibitor) [35] and both genes are strongly induced in HR+ cells at pregnancy day 7 (Figure 4C, 6B and 7C). Secreted SLPI specifically reduces growth of mammary but not colon cancer cells grown in mice [36], suggesting a functional role for SLPI in mammary epithelial cells. The role of the induced SFTPD (Surfactant, pulmonary-associated protein D) seems straight forward; surfactant secretion prevents the collapse of lung alveoli and likely also of the developing mammary alveoli that become apparent by day 7 of pregnancy. In addition, SFTPD is thought to play an important role in innate immunity because it binds a wide variety of microorganisms and may modulate leukocyte responses [37]. Curiously, DMBT1 (Deleted in Malignant Brain Tumors 1) is also expressed in the lung and seems to interact with SFTPD at the protein level [38]. DMBT1 is induced in inflammatory conditions as part of an anti-microbal defense but it is downregulated in the process of terminal differentiation in gastric epithelia [39]. Our data show that DMBT1 is strongly downregulated in HR+ cells in early pregnancy (Figure 7C). In contrast, SFTPD is strongly induced and it is currently unclear whether in mammary alveologenesis DMBT1 and SFTPD are functionally linked. Similar to its downregulation in early pregnancy, DMBT1 expression is also reduced in breast cancer where its expression was anti-correlated with the proliferation marker MCM5 [40]. Thus, the downregulation of DMBT1 in HR+ cells in early pregnancy could be related to an induction of proliferation. Taken together, several of the most strongly induced genes play a role in the protection against microorganisms. Speculatively, there may be an increased risk for a compromised epithelial barrier during active morphogenesis.

Another category of induced genes is involved in cell to cell communication. IGF2 is a prime example of this (Figure 7D), but we also observed the induction of for instance Midkine (MDK) and IL-19 and a strong downregulation of IL-33 (Figure 7D). Like IGF2, Midkine is a growth factor (MDK is also known as neurite growth promoting factor 2) and is widely expressed during embryogenesis but is absent from most adult tissues with the notable exception of the nervous system [41]. We show here that it is just detectable in virgin HR+ cells (average Ct of 33) and strongly induced in pregnancy (average Ct of 28 at day 7, Figure 7C). MDK has been reported to promote proliferation of cancer cells [42]. However, reports about its role in breast cancer are contradictory [43-45] and warrant further investigation of the role of MDK in the different mammary epithelial cell types in normal and malignant mammary development. The cytokine IL-19 was approximately 8-fold upregulated in the biological replicates used for the microarray analysis, but the independent validation by qPCR showed a more moderate response (Figure 7D). It will nevertheless be interesting to further explore the role of IL-19 because it induces proliferation and migration of breast cancer cells and the expression of IL-19 is correlated with a poor clinical outcome [46]. IL-33 is highly expressed in epithelial barrier tissues [47] and thought to act as an ‘alarmin’ that amplifies the innate immune response in case of tissue damage [48]. In contrast to the other genes shown in Figure 7D, IL-33 is abruptly downregulated, possibly to prevent an ‘alarmist’ response in case of alveolar morphogenesis. Preliminary data suggest that IL-33 enhances tumor growth in a mouse model of breast cancer [49] and it will be interesting to investigate the role and cellular target of IL-33 in the mammary gland.

## Conclusion

Pregnancy induces considerable changes in the mammary gland, and many studies have characterized this. Molecular analysis has been primarily performed on entire mammary glands, thereby blending the characteristics of all cell types of the fat pad and all epithelial cells types [50,51]. Sorting mammary epithelial cell populations based on cell surface markers has already provided new insights into cell-type specific transcriptomes of the three epithelial lineages in the adult virgin gland [3,8] and of the involuted mammary gland [52]. Here, we have taken that a step further and analyzed the dynamic transcriptional changes within a specific cell population in early pregnancy. We focussed on the changes in HR+ cells specifically, but similar dynamic changes are likely to occur in the other lineages that contribute to alveologenesis. Our data underscore that HR+ proliferate as part of a normal developmental program that is active in early pregnancy. Estrogen-dependent proliferation is one of the defining features for ER+ breast cancer, however this has been considered a newly acquired trait because ER+ cells in normal non-pregnant breast tissue rarely proliferate. It will be interesting to explore to what extend ER+ cancer cells hijack this developmentally-restricted program.

The transcriptional changes at day 7 of pregnancy show a wide range of responses, part of which seem to involve the increase in anti-microbial defense which might be due to a suboptimal barrier function during active epithelial morphogenesis. Another part of the response at day 7 is likely involved in the coordination of the collaborative outgrowth of different epithelial cell types to form the milk-producing alveoli. Many of the most significantly changed genes have a role in breast cancer and given the heterogeneity within breast tumors, where ER+ breast tumors can contain as few as 10% ER+ cells [53], it will be important to investigate heterotypic signaling in both normal and malignant mammary gland development.

## Methods

### Mice and timed mating

All experiments were conducted with FVB/N mice purchased from Jackson Laboratory and bred and maintained in the animal facility of the DUKE-NUS Graduate Medical School and The National Cancer Centre, Singapore. All animal protocols were approved by the SingHealth Institute Animal Care and Use Committee. The virgin controls were injected with EdU at metestrus when 11 weeks old and mammary glands were harvested 24 hours later. For the early-pregnancy time-points, mice were timed-mated when 9-11 weeks old. Female mice at estrus were placed in the cage of a male after 10 pm and checked for vaginal plugs at 8 am the following morning (Day 0). Mice were injected with EdU 24 hours before euthanizing 3 days or 7 days later by carbon dioxide inhalation. 2 mg/g body weight of EdU (Molecular Probes #C10337) was injected.

### Carmine staining of whole-mounted mammary glands

A #3 (thoracic) gland was fixed in methacarn (60% methanol, 30% chloroform, 10% acetic acid) between glass slides for 24 hours. Subsequently the gland was placed in 70% ethanol for 24 hours, then immersed in 0.2% carmine (Sigma #C1022)- 0.5% aluminum potassium sulfate (Aldrich) stain for 18 hours. Next, glands were transferred to 70%, 90% and 100% ethanol for 1 hour each, followed by 100% ethanol for 18 hours. Finally glands were transferred to methyl salicylate (Sigma #M2047) for visualisation and photography with an Olympus SZX12 microscope.

### Confocal immunofluorescence

Fresh #3 (thoracic) glands were fixed for 18 hours in 4% buffered formaldehyde (ICM Pharma), processed and embedded in paraffin wax. 5 μm sections were cut and adhered to Superfrost Plus coated slides (Menzel-Glaser #J1800AMNZ) overnight at 37°C. Sections were de-paraffinized in Xylene (2× 5 minutes) and hydrated by sequential incubation in ethanol solutions of decreasing concentration (100% ethanol 2× 5 minutes, 90% ethanol 2× 5 minutes, 70% ethanol 2× 5 minutes and distilled H_2_O 5 minutes). Antigen retrieval was performed in 600 mL of 1 mM disodium-EDTA by heating in a microwave on high for 5 minutes and on 30% power for an additional 5 minutes and then cooled at room temperature for 1 hour. Slides were immersed in distilled H_2_O and washed in PBS for 5 minutes. Sections were encircled with a wax pen and incubated for 30 minutes at room temperature with the Click-iT reaction mix (Molecular Probes #C10337), prepared as described by the manufacturer. Primary antibody was diluted in PBS (for dilutions and suppliers, see Additional file 8) with 10% normal serum from the species in which the secondary antibody was raised, was applied and incubated at 4°C overnight. Sections were washed in PBS (3× 5 minutes) before the addition of secondary antibody (in PBS + 10% normal serum), for 30 minutes at room temperature. Sections were washed in PBS (2× 5 minutes) and stained with DAPI (1 μg/mL) for 2 minutes at room temperature. Sections were then washed in PBS and mounted in Vectashield fluorescence mounting media (Vector Laboratories #H-1000). Images were acquired on a Zeiss 710 confocal microscope with a pinhole aperture of 1 airy unit. For cell enumeration, at least 20 fields were randomly selected and greater than 1300 luminal cells counted per animal. Of note, ER protein levels decrease upon pregnancy (see Additional file 3) and to allow accurate identification of ER+ cells for quantification we increased the exposure of samples derived from pregnant animals to a signal that was similar to the samples derived from virgin mice (Figure 1).

### Isolation of primary mammary epithelial cells

Mammary epithelial cells (MECs) were isolated according to [54], with minor modifications [2]. The #4 and #5 mammary glands were excised after removal of mammary lymph nodes and were mechanically and enzymatically digested to single cells. The glands from one animal were pooled and processed as one sample. For more details, see [2]. Single cells were resuspended in L15 medium with 6% FCS, counted and kept on ice during antibody staining for FACS.

### Cell labeling, flow cytometric analysis & fluorescence-activated cell sorting (FACS)

Fluorochrome-conjugated antibodies were titrated on primary mammary epithelial cells to ensure maximal positive:background fluorescence ratio (Additional file 9). Anti-mouse &/or anti-rat compensation beads (BD #552843 and #552845, respectively) were used for single stain antibody controls. Compensation controls also included two cellular samples: unstained cells and cells with DAPI (Sigma #D8417). Single cell samples from individual animals (3 animals per time point) were incubated with antibodies on ice for 45 minutes with agitation each 15 minutes. Samples were then washed with twice the sample volume and resuspended in L15+ containing 200 ng/mL of DAPI, except non-DAPI compensation controls. All multiple-labelled samples were gated on FSC-A vs. SSC-A and doublet discrimination (FSC-H vs. FSC-W & SSC-H vs. SSC-W) and DAPI negativity (Additional file 1). Samples contained anti-CD45 to exclude lymphocytes from analysis. Cells were analyzed and sorted on a BD FACS-Aria II containing 355 nm UV, 488 nm blue, 561 nm yellow-green and 633 nm red lasers. An overview of the FACS proportions of the individual animals used for this study can be found in Additional file 2.

### Generation of cDNA by direct reverse transcription & qPCR analysis

For analysis of transcript levels by qPCR, cells were sorted directly into lysis buffer (10 IU RNase inhibitor (Invitrogen), 2 mM DTT, 0.15% Tween-20 (Biorad) in 10 μL of nuclease-free water) in PCR tubes. 500 cells were sorted into each tube, making approximately 12 μL total volume. Reverse transcription was performed using Superscript VILO (Invitrogen #11754) as per manufacturers protocol. Primers were designed that span introns to exclude the detection of genomic DNA and selected for optimum melt curve and amplification profiles (for primer sequences, see Additional file 9). qPCR was performed using SSo Fast Evagreen supermix reagent (Biorad #172-500) as per manufacturers protocol. Per condition 3 animals were assayed, normalized by HPRT (validated to be consistent between groups), averaged and compared to Virgin samples according to the delta-delta c (t) method. The relative values from 3-5 sets of mice were assessed by paired *t*-test for statistical significance.

### Preparation of RNA for the microarray

For Microarray analyses, cells were sorted directly into lysis buffer (20 IU RNase inhibitor (Invitrogen), 2 mM DTT, 0.15% Tween-20 (Biorad) in 8 μL of nuclease-free water) in PCR tubes. 1000 cells were sorted into each tube, making approximately 12 μL total volume. The cells were allowed to lyse for 15 minutes on ice and stored at -80 degrees. RNA from 2 μL of lysed cells (equivalent of 167 cells) was amplified and labelled with Biotin using the TargetAmp™ 2-Round Biotin-aRNA Amplification Kit 3.0 (epicentre #TAB2R710) as per the manufacturers protocol. Sorting a thousand cells improves the representation of the HR+ population and the accuracy of the sort (because of the collection volume of 8 μL). The maximum input in the TargetAmp Kit was 2 μL and we used the leftover RNA for quality controls. The TargetAmp output was subjected to SuperScript® II Reverse Transcriptase (Invitrogen #18064) and SuperScript® III Reverse Transcriptase (Invitrogen #180800) to synthesize cDNA and RNA Clean & Concentrator™(ZYMO Research #R1017) and RNeasy MinElute Cleanup Kit (Qiagen #74204) were used to purify the RNA as recommended by the amplification protocol. The concentration and purity of the Biotin-labelled aRNA was determined by using a NanoDrop spectrophotometer (Thermo Scientific-NanoDrop 2000).

### Microarray analysis

Microarray hybridization and scanning was performed by the Genomic and RNA Profiling Core Facility at DUKE-NUS Graduate Medical School. 1.5 μg of biotinylated a/cRNA from each sample was hybridized at 58°C for 16 hours using the Illumina Whole-Genome Gene Expression Direct Hybridization Assay system with the Illumina Mouse WG-6 v2.0 (six-sample BeadChip) platform. The signal was developed using streptavidin-Cy3 and the BeadChips were scanned with an Illumina BeadArray Reader. Raw signals were logarithmically transformed (to base 2) and quantile normalized. The background noise from the array was determined at 100, and probes with an average signal <100 in all comparator groups were removed from further analysis (this reduced the number of analyzed probes from 45281 to 19821). Differential gene expression between pregnant and virgin samples was expressed as a log ratio of the average logged signals between the compared groups. Statistical significance of differential gene expression was assessed by a regularized *t*-test adapted for small replication groups (Cyber-T, [55]). False discovery rates (FDR) were generated on the nominal p-values by the multiple testing correction procedure of Benjamini and Hochberg [56]. Generally, genes with an absolute fold-change of 2-fold or higher and a FDR less than or equal to 10% were considered to be significantly and differentially expressed and formed the basis for comparing gene lists from each comparison (3D vs Vir and 7D vs Vir). For each probe, a combined metric (Pi score) based on the signed log ratio and the FDR was additionally defined [57] and computed as follows: Pi = (log ratio)*(-log10 [FDR]). The Pi score was used to determine the top 25 up- and down-regulated genes in the 3-day and 7-day pregnant samples compared to the virgin group. Principal components analysis was conducted on the expression covariance matrix to identify potential sample outliers (Partek Genomics Suite, version 6.6). The microarray data from this publication have been submitted to the NCBI Gene Expression Omnibus and are deposited as GSE63720.

### Pathway enrichment analysis

Pathway enrichment analysis was conducted via the over-representation analysis (ORA) method in the Ingenuity Pathway Analysis (IPA) tool (Ingenuity Systems, www.ingenuity.com). A pre-filtered list of differentially expressed genes (absolute fold-change ≥ 2-fold and FDR ≤ 10%) were used as input for each comparison. Pathway enrichment was assessed on the list of canonical pathways from the Ingenuity Knowledge Base. Over-representation of biological pathways was ascertained via Fisher’s exact test and corrected for multiple testing by the Benjamini-Hochberg procedure.

### Declaration of compliance with guidelines for the use of animals in this study

All procedures were performed in accordance with the guidelines for ethical treatment of laboratory animals approved by the SingHealth Institute Animal Care and Use Committee, Singapore. FVB/N mice were purchased from Jackson Laboratory and bred and maintained in the animal facility of the DUKE-NUS Graduate Medical School and The National Cancer Centre, Singapore under guidelines for housing and husbandry conditions of each institute. Mice were euthanized by carbon dioxide inhalation and cervical dislocation. Effort was taken to ensure good animal welfare and prevent suffering.

### Availability of supporting data

The data set supporting the results from this article is available in the NCBI Gene Expression Omnibus, GSE63720.

## Additional files

Additional file 1:
**Gating strategy used for fluorescence-activated cell sorting (FACS).** Cell doublets and debris were excluded from the murine mammary gland cell suspension using the Forward and Side scatter parameters. Single viable cells were gated using DAPI before excluding lymphocytes using CD45. CD24 and CD49f were used to subdivide the epithelial cells into Luminal and Basel lineages. The luminal population was further subdivided into the HR+ and Alveolar cell populations using Sca1 and CD49b expression. Additional file 2:
**FACS proportions of mammary epithelial subpopulations of the samples used for the microarray.** (A) Proportion of basal (blue) and luminal (green) epithelial cells based on FACS using cell surface markers CD24 and CD49f for cells isolated from 3 virgin mice (Vir-1, Vir-2 and Vir-3), 3 mice that were 3-days pregnant (3D-1, 3D-2 and 3D-3) and 3 mice that were 7 days pregnant (7D-1, 7D-2 and 7D-3). (B) Proportion of hormone receptor positive (HR+, purple) and alveolar progenitor (yellow) luminal cells based on FACS using cell surface markers Sca1 and CD49b for the same samples shown in (A). Additional file 3:
**Immuno-fluorescence staining illustrating the reduction of hormone receptor expression during pregnancy.** Estrogen Receptor (ER, green), Progesterone Receptor (PR, red), Cyto-keratin 8 (CK8, blue) and DAPI (Grey). Scale bar, 10 μm. Images were acquired with a fixed exposure time. Additional file 4:
**List of genes that significantly change in hormone-sensing cells at day 3 and day 7 of pregnancy.** (A) List of 165 genes differentially expressed in HR+ cells at day 3 of pregnancy compared to virgin animals based on Venn analysis conducted on a list of genes that had an absolute fold-change of > = 2-fold and a FDR of 10% or less. Since some genes are represented by multiple Illumina probes, the analysis was conducted at the gene symbol level. (B) List of 401 genes differentially expressed in HR+ cells at both day 3 and 7 of pregnancy compared to virgin animals based on Venn analysis. (C) List of 605 genes differentially expressed in HR+ cells at day 7 of pregnancy compared to virgin animals based on Venn analysis. Additional file 5:
**Enrichment for gene function by Ingenuity Pathway Analysis (IPA) systems.** List of genes belonging to canonical Ingenuity pathways that were differentially expressed in HR+ cells in early pregnancy. Pathway enrichment analysis was conducted via over-representation analysis in Ingenuity Pathway Analysis software, using a pre-filtered list of differentially expressed genes (absolute fold-change ≥ 2-fold and FDR ≤ 10%) as the input. Pathway significance was ascertained by the right-tailed Fisher’s exact test (column B) and the ratio of the number of pathway-specific differentially expressed genes to the total number of genes for the pathway are shown in column C. Columns D-O list the individual differentially expressed genes for each pathway. Additional file 6:
**Cell cycle regulators identified in HR+ cells in early pregnancy.** (A) Fold change of Log 2 expression of pre-selected cell cycle genes at pregnancy day 3 and 7 compared to virgin samples. At 3 days of pregnancy, up-regulated genes are indicated in red, down-regulated genes in green and unchanged genes in grey. (B) Schematic representation of pre-selected genes involved in cell cycle regulation at 3 days of pregnancy. Up-regulated genes are indicated in red, down-regulated genes in green and unchanged genes in grey. Additional file 7:
**List of genes encoding secreted factors that are differentially expressed in HR+ cells at day 7 of pregnancy.** (A) List of genes encoding secreted factors differentially expressed at day 7 of pregnancy compared to virgin animals. Data was generated by cross-comparing the genes of interest against the master list of Uniprot proteins with subcellular location = ‘secreted’ (confidence level = any). This list corresponds to the Gene Ontology term “extracellular region (GO0005576)”. (B) Top 50 up-regulated genes encoding secreted factors at day 7 of pregnancy. (C) Top 50 down-regulated genes encoding secreted factors at day 7 of pregnancy. Additional file 8:
**Antibodies used in confocal immunofluorescence and fluorescence-activated cell sorting (FACS).**
Additional file 9:
**Nucleic acid sequences for primers used in Quantitative polymerase chain reaction (qPCR) experiments.**

## Abbreviations

HR: Hormone receptor
IGF2: Insulin-like growth factor 2
ER: Estrogen receptor
PR: Progesterone receptor
Elf5: E74-like factor 5
RANKL: Receptor activator of nuclear factor kappa-B ligand, also known as TNFSF11
FACS: Fluorescence-activated cell sorting
qPCR: Quantitative polymerase chain reaction
RT: Reverse transcriptase
EDTA: Ethylenediaminetetraacetic acid
EdU: 5-Ethynyl-2’-deoxyuridine
CK8: Cytokeratin-8
DAPI: 4’,6-Diamidino-2-phenylindole
FDR: False discovery rate
GSEA: Gene-set enrichment analysis
PCA: Principal component analysis
IGFALS: Insulin-like growth factor acid labile subunit
IGFBP: IInsulin-like growth factor binding protein
CDCa3: Cell division cycle associated 3
CDCa8: Cell division cycle associated 8
PBK: PDZ-binding kinase
Kif22: Kinesin-like protein 22
KNTC1: Kinetochore associated 1
Nusap1: Nucleolar and spindle-associated protein 1
MCM5: Minichromosome maintenance complex component 5
CALCA: Calcitonin
IDO1: Indoleamine 2,3 dioxygenase
Wnt4: Wingless-type MMTV integration site family, member 4
SLC5A5: Solute carrier family 5
DBP: D site of albumin promoterbinding protein
WFDC: WAP four-disulfide core domain
SFTPD: Surfactant, pulmonary-associated protein D
DMBT1: Deleted in Malignant Brain Tumors 1
MDK: Neurite growth promoting factor 2
IL-19: Interleukin 19
IL-33: Interleukin 33
