# Supplementary figures and images for: Transcriptome analysis of the hormone-sensing cells in mammary epithelial reveals dynamic changes in early pregnancy
Source: BMC Dev Biol. 2015 Jan 27;15:7. doi: 10.1186/s12861-015-0058-9 (PMC4314744; doi:10.1186/s12861-015-0058-9)

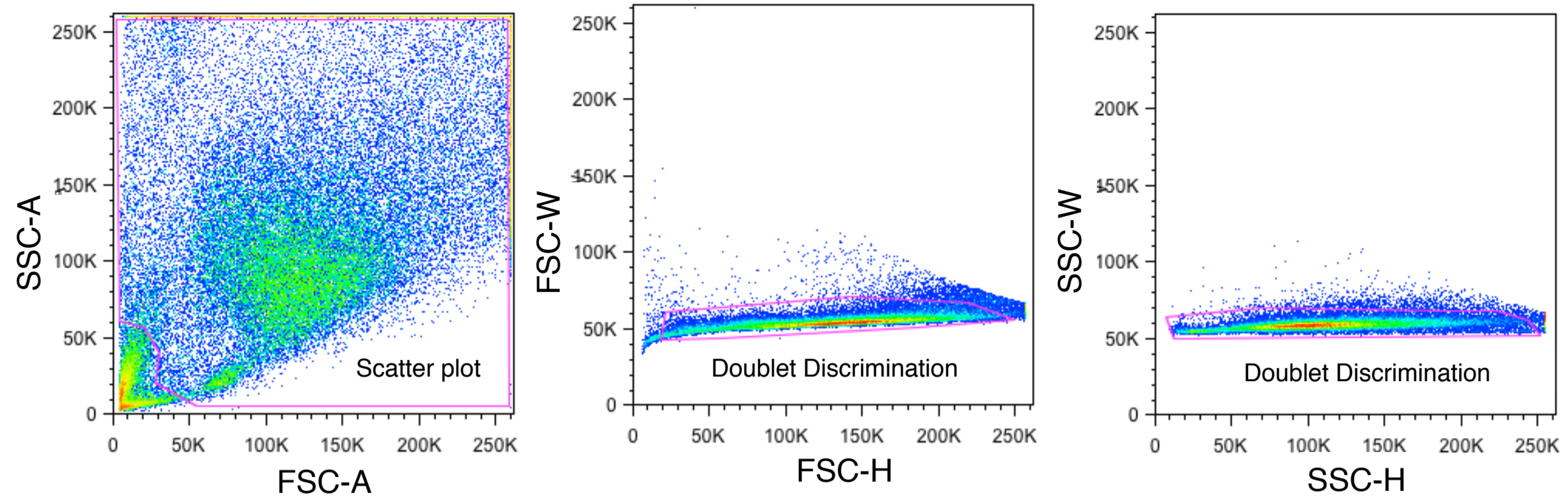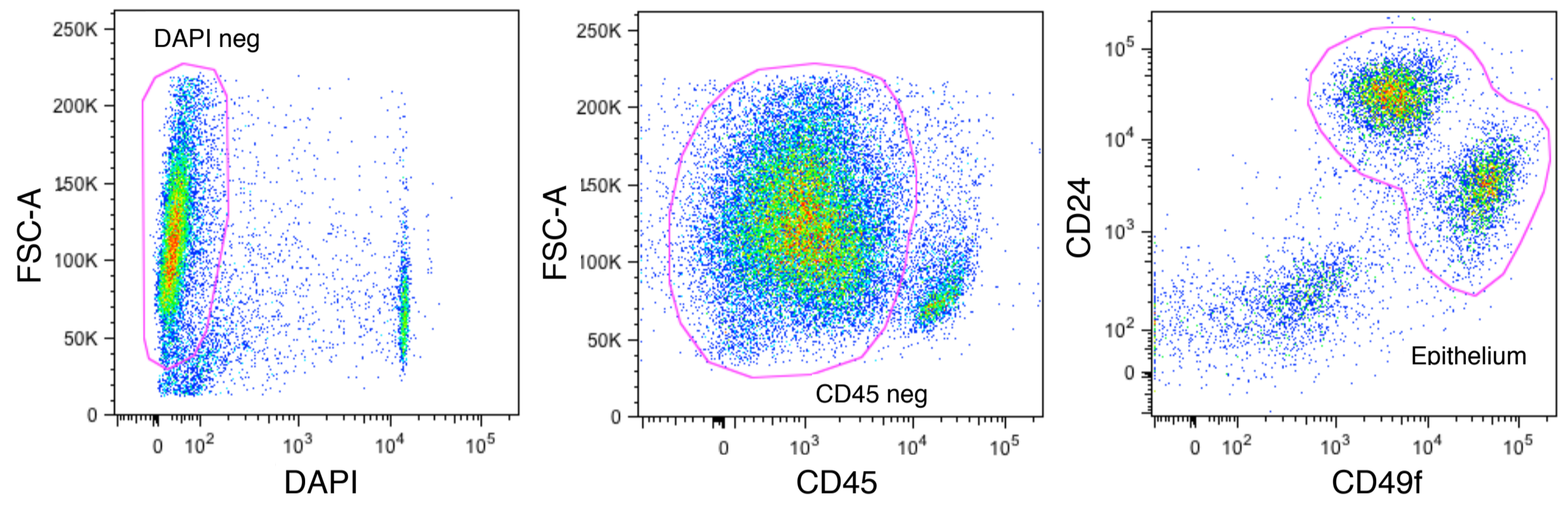

**Virgin**

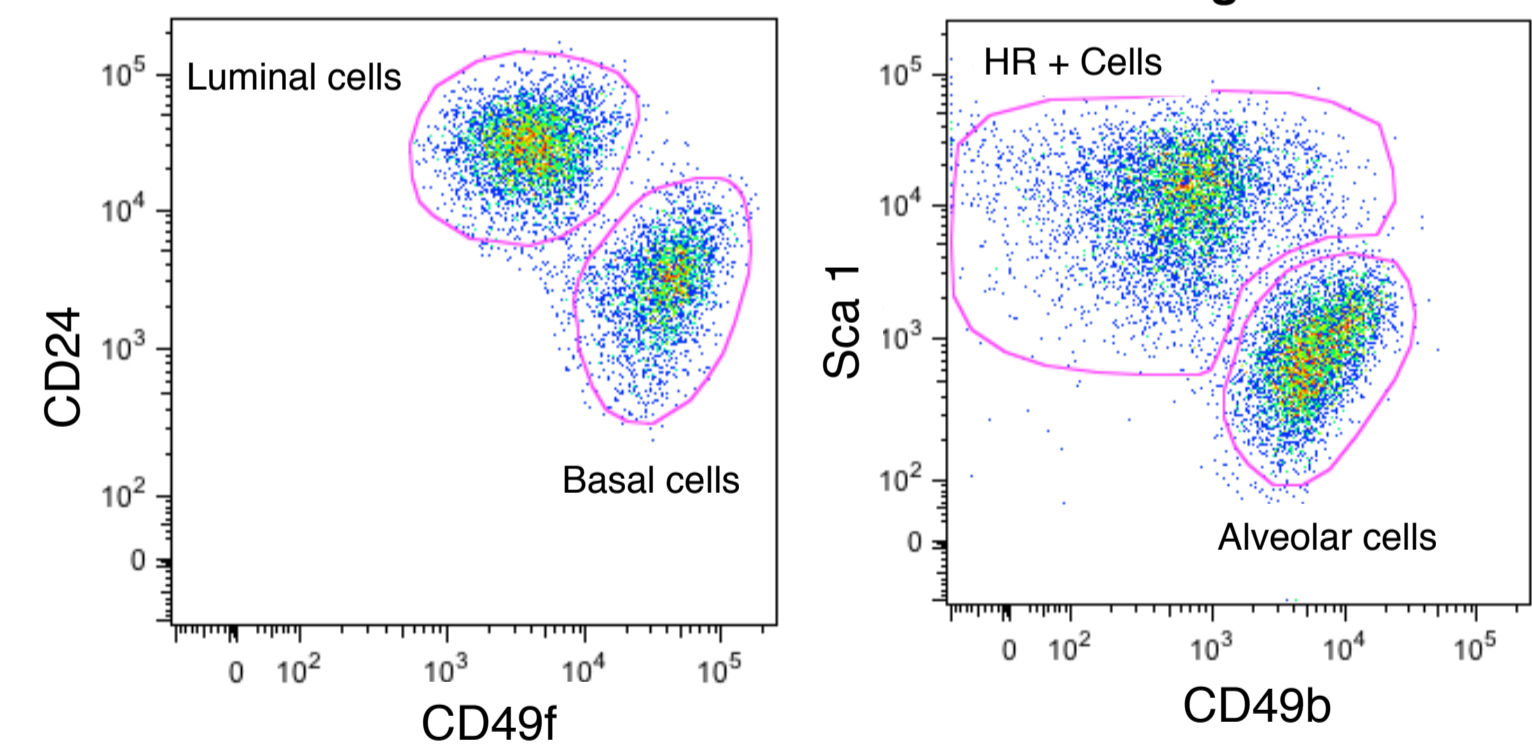

**3d pregnant**

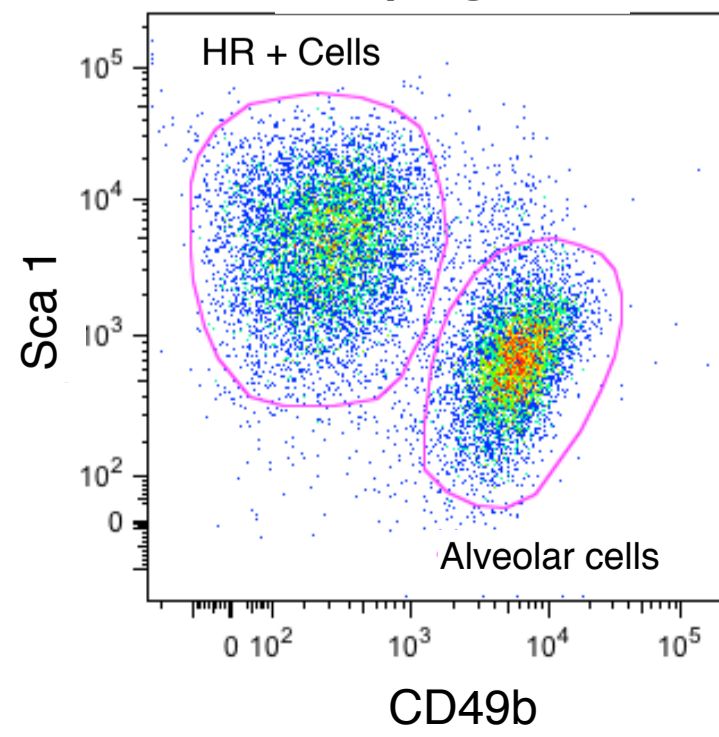

**7d pregnant**

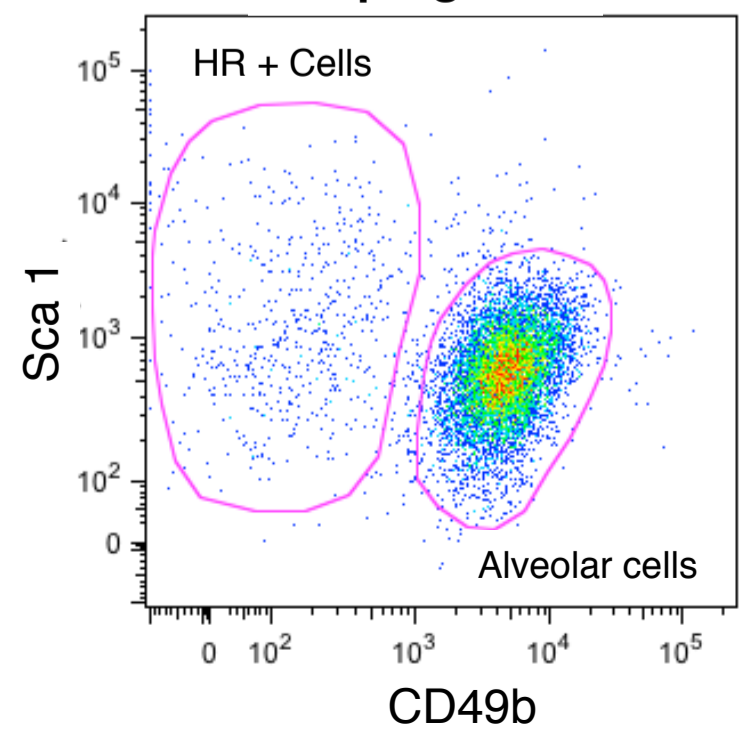

Supplement: Additional file 1: — Gating strategy used for fluorescence-activated cell sorting (FACS). Cell doublets and debris were excluded from the murine mammary gland cell suspension using the Forward and Side scatter parameters. Single viable cells were gated using DAPI before excluding lymphocytes using CD45. CD24 and CD49f were used to subdivide the epithelial cells into Luminal and Basel lineages. The luminal population was further subdivided into the HR+ and Alveolar cell populations using Sca1 and CD49b expression. [file 12861_2015_58_MOESM1_ESM.pdf]

A

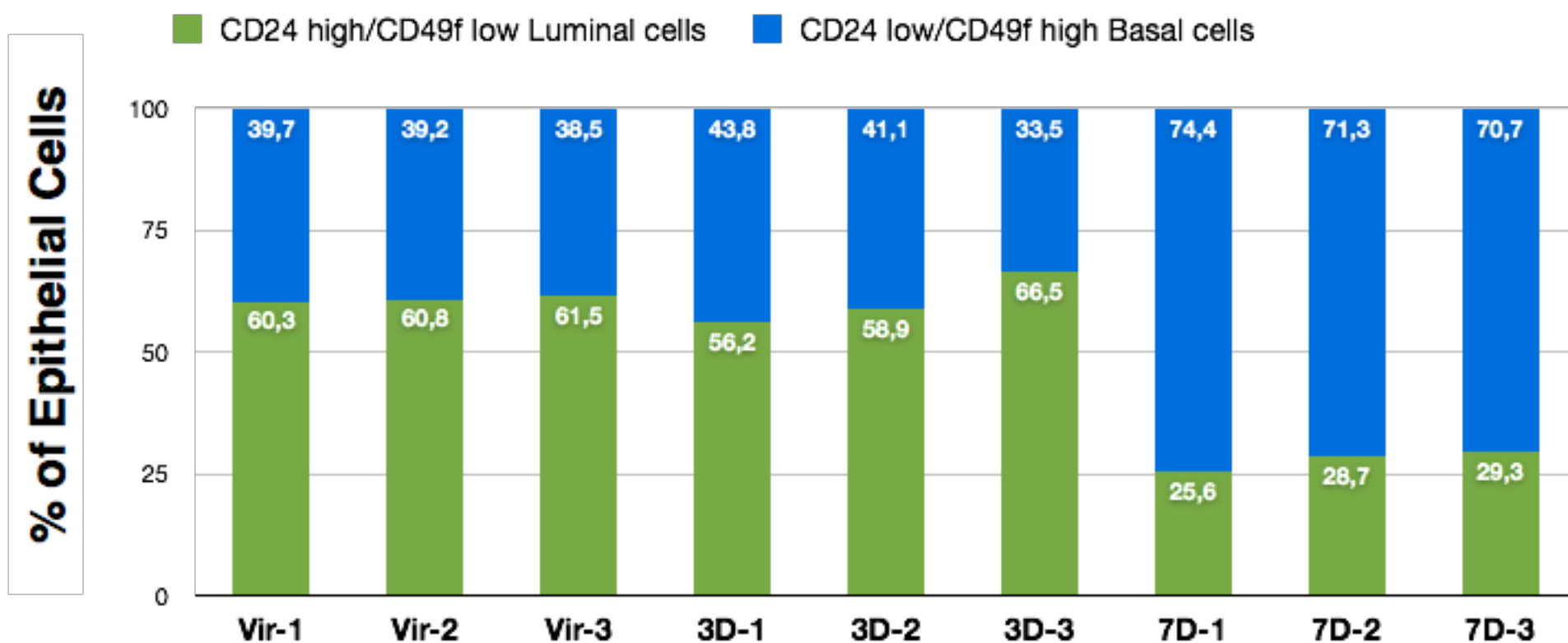

B

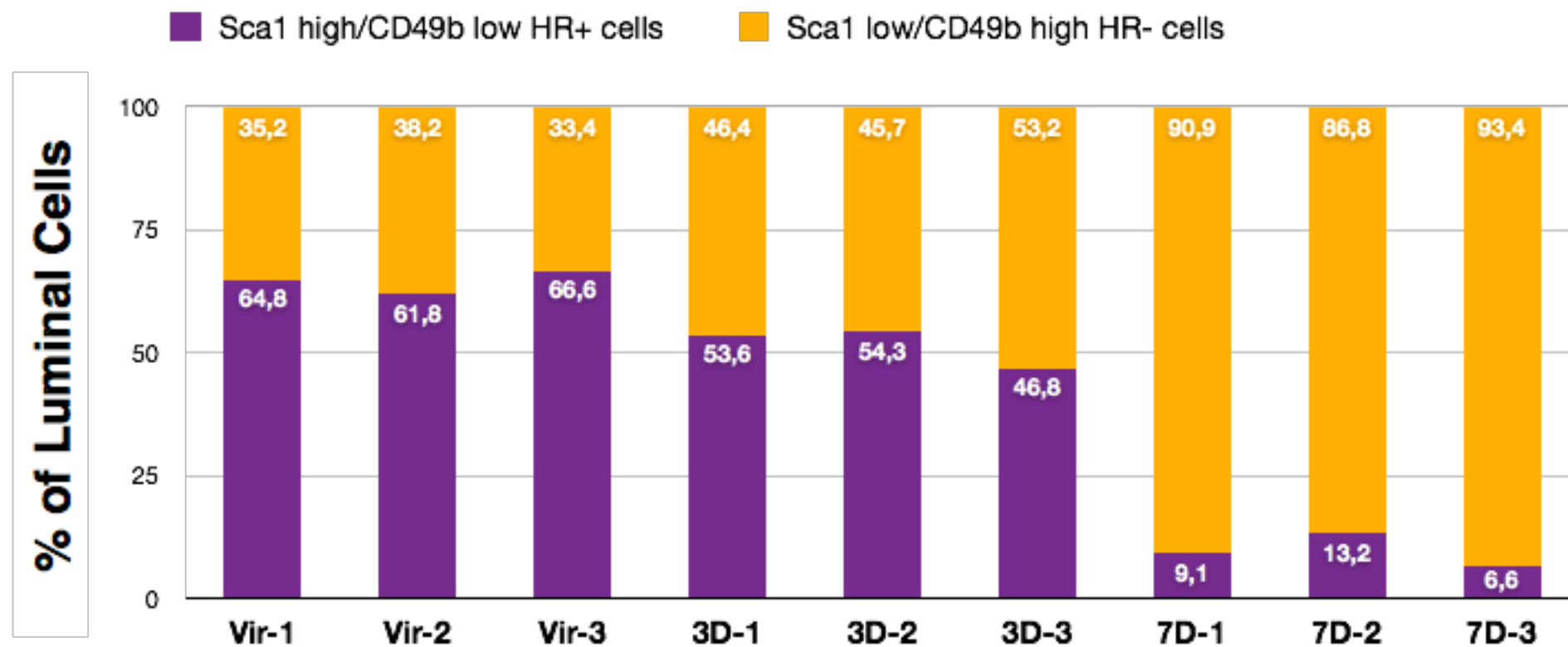

Supplement: Additional file 2: — FACS proportions of mammary epithelial subpopulations of the samples used for the microarray. (A) Proportion of basal (blue) and luminal (green) epithelial cells based on FACS using cell surface markers CD24 and CD49f for cells isolated from 3 virgin mice (Vir-1, Vir-2 and Vir-3), 3 mice that were 3-days pregnant (3D-1, 3D-2 and 3D-3) and 3 mice that were 7 days pregnant (7D-1, 7D-2 and 7D-3). (B) Proportion of hormone receptor positive (HR+, purple) and alveolar progenitor (yellow) luminal cells based on FACS using cell surface markers Sca1 and CD49b for the same samples shown in (A). [file 12861_2015_58_MOESM2_ESM.pdf]

Virgin

3D Pregnant

7D Pregnant

CK8 ER PR

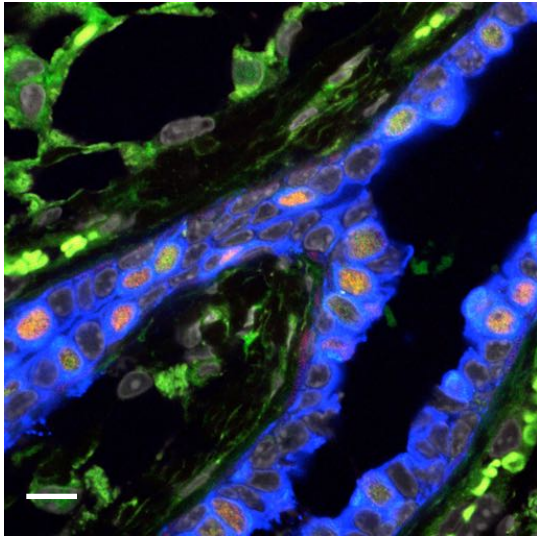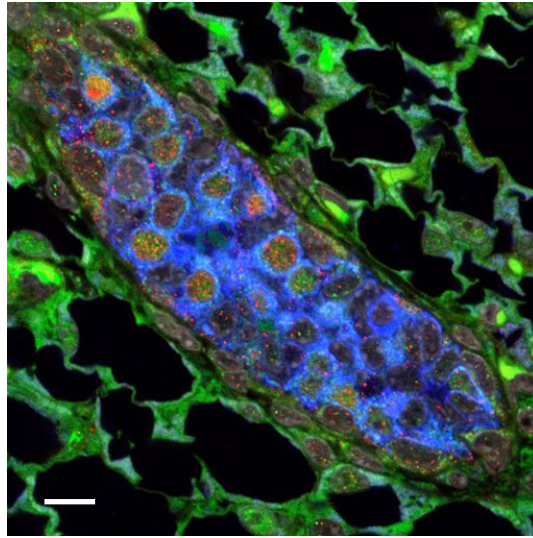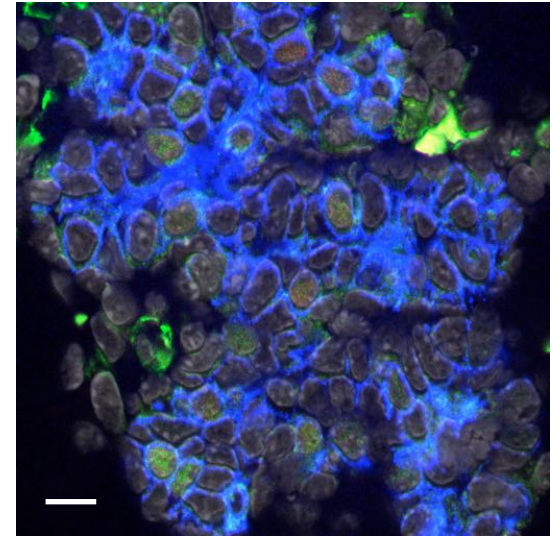

CK8 ER

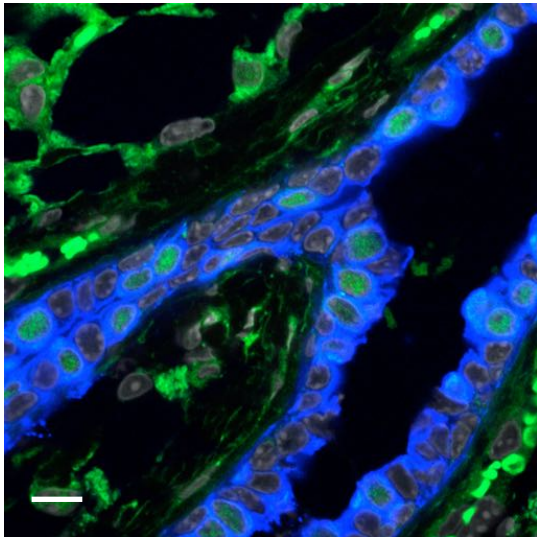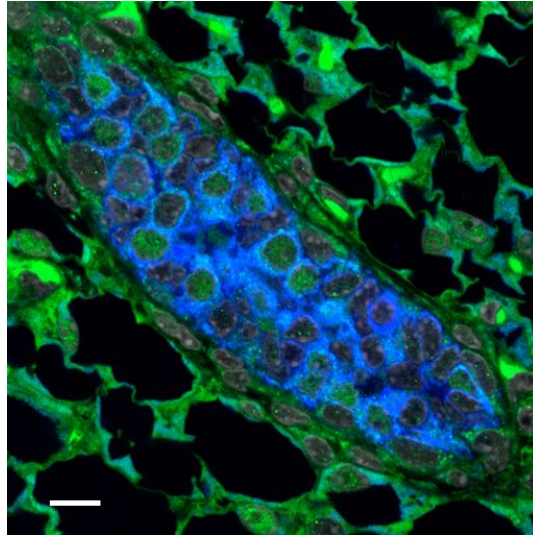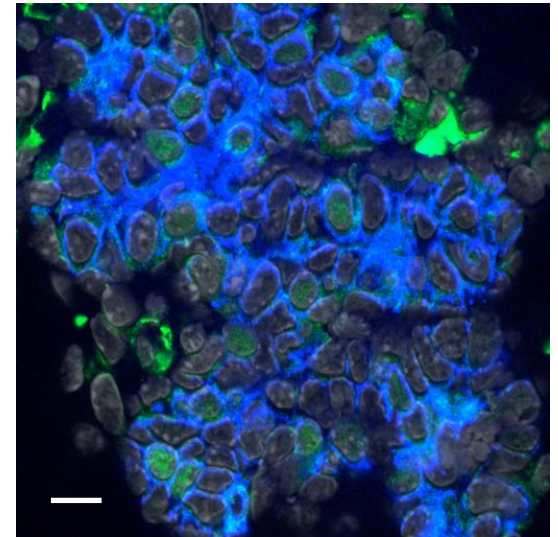

CK8 PR

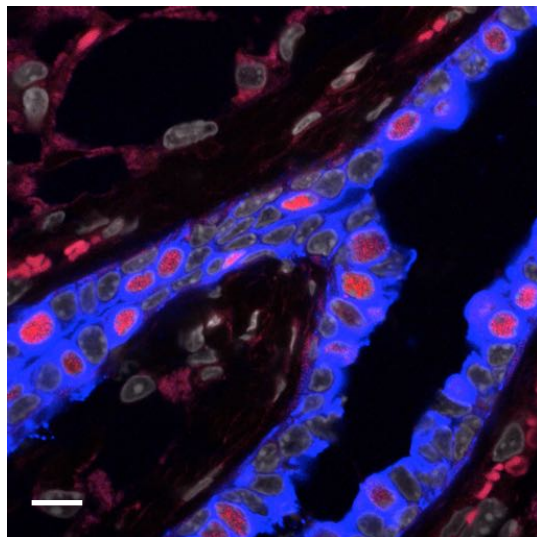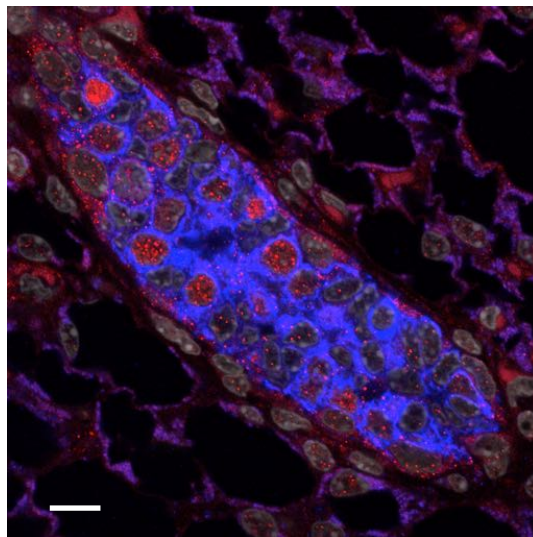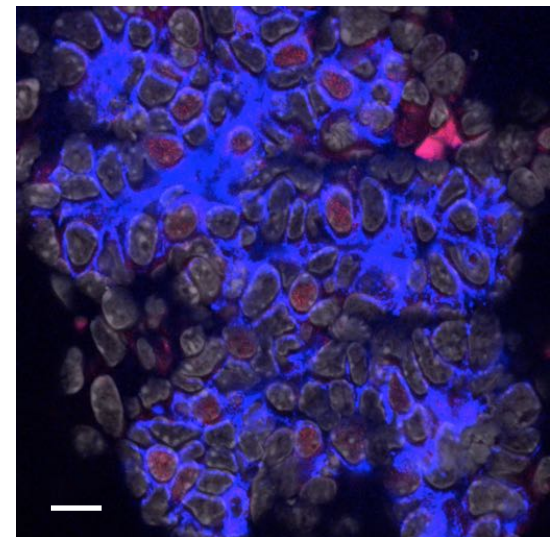

Supplement: Additional file 3: — Immuno-fluorescence staining illustrating the reduction of hormone receptor expression during pregnancy. Estrogen Receptor (ER, green), Progesterone Receptor (PR, red), Cyto-keratin 8 (CK8, blue) and DAPI (Grey). Scale bar, 10 μm. Images were acquired with a fixed exposure time. [file 12861_2015_58_MOESM3_ESM.pdf]
