# Supplementary material for: Transcriptome analysis of the hormone-sensing cells in mammary epithelial reveals dynamic changes in early pregnancy
Source: BMC Dev Biol. 2015 Jan 27;15:7. doi: 10.1186/s12861-015-0058-9 (PMC4314744; doi:10.1186/s12861-015-0058-9)

A

	3D vs Vir	7D vs Vir
**Cdk4**	**1.77**	1.18
**Cdk6**	-0.10	-0.14
**CyclinD1**	0.04	-0.61
**Cdk2**	0.22	-0.20
**Cyclin E**	0.21	0.09
**p15**	**-0.79**	-1.51
**p16**	0.05	-0.02
**p19**	-0.09	-0.00
**p21**	**-1.71**	-1.72
**p27**	-0.10	-0.12
**Cdc25a**	-0.01	0.02
**E2f1**	**2.34**	0.365
**E2f2**	0.05	0.10
**E2f3**	**0.45**	0.44
**E2f4**	**0.48**	0.25
**E2f5**	-0.33	-0.12
**E2f6**	0.15	-0.01
**E2f7**	0.12	0.02
**Cdk2**	0.22	-0.20
**Cyclin A**	0.17	0.01
**CDK1**	0.38	-0.16
**Cdt1**	**2.41**	0.62
**Cdc6**	0.05	0.16
**Cyclin B**	**3.13**	1.00
**Pbk**	**5.58**	2.36
**Cdc25c**	**2.09**	0.76
**Plk1**	**2.30**	0.52
**Wee1**	**-0.42**	-0.32
**Myt1**	**1.75**	3.15
**Aurka**	**1.68**	0.27

B

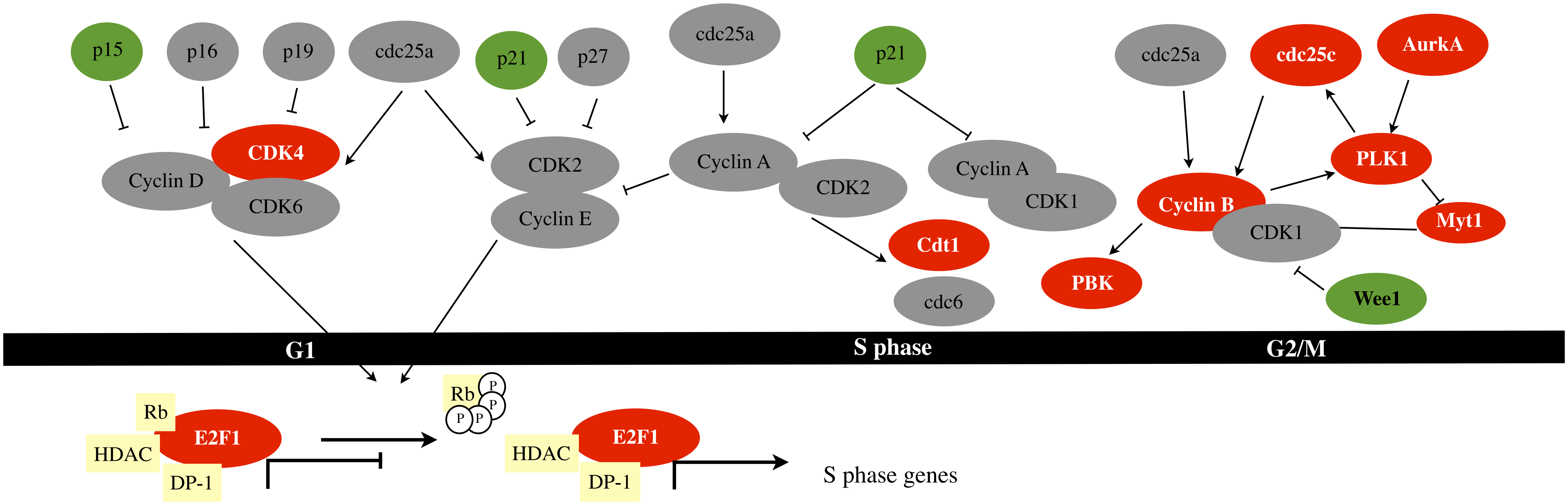

Supplement: Additional file 6: — Cell cycle regulators identified in HR+ cells in early pregnancy. (A) Fold change of Log 2 expression of pre-selected cell cycle genes at pregnancy day 3 and 7 compared to virgin samples. At 3 days of pregnancy, up-regulated genes are indicated in red, down-regulated genes in green and unchanged genes in grey. (B) Schematic representation of pre-selected genes involved in cell cycle regulation at 3 days of pregnancy. Up-regulated genes are indicated in red, down-regulated genes in green and unchanged genes in grey. [file 12861_2015_58_MOESM6_ESM.pdf]
