# Supplementary material for: Transcriptome analysis of the hormone-sensing cells in mammary epithelial reveals dynamic changes in early pregnancy
Source: BMC Dev Biol. 2015 Jan 27;15:7. doi: 10.1186/s12861-015-0058-9 (PMC4314744; doi:10.1186/s12861-015-0058-9)
Supplement: Additional file 8: — Antibodies used in confocal immunofluorescence and fluorescence-activated cell sorting (FACS). [file 12861_2015_58_MOESM8_ESM.pdf]

Antigen	Species	Dilution	Supplier	Cat #	Conjugate
Cytokeratin 8	Rat	1:100	Developmental Studies Hybridoma Bank	TROMA-1	
Estrogen Receptor	Mouse	1:100	Novacastra	NCL-ER-6F11	
CD45	Rat	1:500	Becton Dickinson	550994	PerCP-Cy5.5
CD24	Rat	1:250	Becton Dickinson	553261	FITC
CD49f	Rat	1:500	Becton Dickinson	555736	PE-Cy5
Sca 1	Rat	1:166	Becton Dickinson	558162	PE-Cy7
CD49b	Hamster	1:250	Becton Dickinson	558759	PE
