# Supplementary material for: Transcriptome analysis of the hormone-sensing cells in mammary epithelial reveals dynamic changes in early pregnancy
Source: BMC Dev Biol. 2015 Jan 27;15:7. doi: 10.1186/s12861-015-0058-9 (PMC4314744; doi:10.1186/s12861-015-0058-9)
Supplement: Additional file 9: — Nucleic acid sequences for primers used in Quantitative polymerase chain reaction (qPCR) experiments. [file 12861_2015_58_MOESM9_ESM.pdf]

Mouse Gene ID	Sequence 5'-3'
HPRT (F)	CTGGTGAAAAGGACCTCTCG
HPRT (R)	TGAAGTACTCATTATAGTCAAGGGCA
Estrogen Receptor (F)	GCCAAGGAGACTCGCTACTG
Estrogen Receptor (R)	CTCCGGTTCTTGTCAATGGT
Prgesterone Receptor (F)	GGTGGAGGTCGTACAAGCAT
Prgesterone Receptor (R)	CTCATGGGTCACCTGGAGTT
Elf5 (F)	GGACTCCGTAACCCATAGCA
Elf5 (R)	TACTGGTCGCAGCAGAATTG
Sca1 (F)	TGCAACCTTGTCTGAGAGGA
Sca1 (R)	TGGGACTCCATAGCACTGGT
Mcm5 (F)	CCATTGCCTGCCTGCTTTTT
Mcm5 (R)	GACTTGGCTGTACCAGGGTC
Bub1 (F)	GGAGATCCGAGCTGAAGTGT
Bub1 (R)	TGAACAGCCTGCATTGCCTT
Cdca 3 (F)	CAGCTGAACGGTCTCAAACA
Cdca 3 (R)	TGGTCCTCAGGGGATAACTG
Kntc1 (F)	TGTCCGTCGGGAAGAAGTGT
Kntc1 (R)	CTTGAGCCAACACTCAGGCA
Calcitonin (F)	AAGAAGAAGTTCGCCTGCTG
Calcitonin (R)	CCAGCATGCAGGTACTCAGA
RANKL (F)	CCCACAATGTGTTGCAGTTC
RANKL (R)	TCCTGAGACTCCATGAAAACG
Wnt 4 (F)	ACTGGACTCCCTCCCTGTCT
Wnt 4 (R)	GGACGTCCACAAAGGACTGT
Cyclin D1 (F)	GGGTGGGTTGGAAATGAACT
Cyclin D1 (R)	CTTCCTCTCCAAAATGCCAG
Wfdc (F)	TGGAGTCAAAGGCGAGGAGA
Wfdc (R)	GCACGCATTTGAAGCCACAT
Slpi (F)	GGACTGTGGAAGGAGGCAAA
Slpi (R)	CCCAGTCAGTACGGCATTGT
Sftpd (F)	GTAGCCCAACAGAGAATGGC
Sftpd (R)	GAGAGCCCCATAGGTCCTG
Dmbt (F)	CCGGCACAATGGGGATCT
Dmbt (R)	AGAATCTGTTCCATCTGTGGGA
IGF2 (F)	GTCGATGTTGGTGCTTCTCA
IGF2 (R)	AAGCAGCACTCTTCCACGAT
Mdk (F)	AGACCATCCGCGTGACTAAG
Mdk (R)	GCTCTCTGGCCTCCTGACT
IL-19 (F)	ATCCTGTCCCTGGAGAACCT
IL-19 (R)	AAAGAGTTGGCAATGCTGCT
IL-33 (F)	ATTTCCCCGGCAAAGTTC
IL-33 (R)	CTTATGGTGAGGCCAGAACG
